# Dissecting the MUC5AC/ANXA2 signaling axis: implications for brain metastasis in lung adenocarcinoma

**DOI:** 10.1038/s12276-024-01255-6

**Published:** 2024-06-03

**Authors:** Sanjib Chaudhary, Jawed Akhtar Siddiqui, Muthamil Iniyan Appadurai, Shailendra Kumar Maurya, Swathi P. Murakonda, Elizabeth Blowers, Ben J. Swanson, Mohd Wasim Nasser, Surinder K. Batra, Imayavaramban Lakshmanan, Apar Kishor Ganti

**Affiliations:** 1 Department of Biochemistry and Molecular Biology, University of Nebraska Medical Center, Omaha, NE 68198-5870 USA; 2 Fred & Pamela Buffett Cancer Center University of Nebraska Medical Center, Omaha, NE 68198-5870 USA; 3 Department of Internal Medicine, University of Nebraska Medical Center, Omaha, NE 68198-1850 USA; 4 Department of Pathology and Microbiology, University of Nebraska Medical Center, Omaha, NE 68198-1850 USA; 5 Division of Oncology-Hematology, Department of Internal Medicine, VA Nebraska Western Iowa Health Care System, Omaha, NE 68105-1850 USA

**Keywords:** Oncogenesis, Non-small-cell lung cancer

## Abstract

Non-small cell lung carcinoma (NSCLC) exhibits a heightened propensity for brain metastasis, posing a significant clinical challenge. Mucin 5ac (MUC5AC) plays a pivotal role in the development of lung adenocarcinoma (LUAD); however, its role in causing brain metastases remains unknown. In this study, we aimed to investigate the contribution of MUC5AC to brain metastasis in patients with LUAD utilizing various brain metastasis models. Our findings revealed a substantial increase in the MUC5AC level in LUAD brain metastases (LUAD-BrM) samples and brain-tropic cell lines compared to primary samples or parental control cell lines. Intriguingly, depletion of MUC5AC in brain-tropic cells led to significant reductions in intracranial metastasis and tumor growth, and improved survival following intracardiac injection, in contrast to the observations in the control groups. Proteomic analysis revealed that mechanistically, MUC5AC depletion resulted in decreased expression of metastasis-associated molecules. There were increases in epithelial-to-mesenchymal transition, tumor invasiveness, and metastasis phenotypes in tumors with high MUC5AC expression. Furthermore, immunoprecipitation and proteomic analysis revealed a novel interaction of MUC5AC with Annexin A2 (ANXA2), which activated downstream matrix metalloproteases and facilitated extracellular matrix degradation to promote metastasis. Disrupting MUC5AC-ANXA2 signaling with a peptide inhibitor effectively abrogated the metastatic process. Additionally, treatment of tumor cells with an astrocyte-conditioned medium or the chemokine CCL2 resulted in upregulation of MUC5AC expression and enhanced brain colonization. In summary, our study demonstrates that the MUC5AC/ANXA2 signaling axis promotes brain metastasis, suggesting a potential therapeutic paradigm for LUAD patients with high MUC5AC expression.

## Targeting MUC5AC/ANXA2 axis for lung adenocarcinoma brain metastasis

Lung cancer frequently moves to the brain, but why is unclear. Scientists have found that a protein, MUC5AC, is crucial in this. The research, led by Sanjib Chaudhary and team, discovered that MUC5AC works with another protein, ANXA2, to help lung cancer cells move to the brain. They also found that astrocytes (a type of brain cell), release a substance that boosts the presence of MUC5AC in lung cancer cells. This research was a lab experiment using lung cancer cells and mice. They found that lowering MUC5AC in lung cancer cells greatly reduced their movement to the brain in mice. This suggests that focusing on MUC5AC could help stop lung cancer from moving to the brain. Future studies will need to confirm these results and look into possible treatments.

This summary was initially drafted using artificial intelligence, then revised and fact-checked by the author.

## Introduction

Brain metastasis is a common occurrence in various cancers and affects ~16–20% of lung cancer (LC) patients during the course of their disease^1–4^. Concerningly, ~30% of LC patients succumb to brain metastasis^3,5^. The underlying reasons that certain tumors exhibit a higher propensity for brain metastasis remain undetermined. The incidence of brain metastasis in LC patients increases to 50–60% with a gene mutation in oncogenic drivers such as the epidermal growth factor receptor and translocations in anaplastic lymphoma kinase^5,6^. Given that most treatment modalities for brain metastases are palliative^7^, these patients typically survive for less than seven months after diagnosis^8^. The increasing incidence and high mortality of brain metastasis underscore the urgent need to identify prognostic markers and druggable targets, necessitating a deeper cellular and molecular understanding of the disease.

In normal lung physiology, mucins (MUCs), particularly Mucin 5ac (MUC5AC) and Mucin 5B (MUC5B), play a vital role in maintaining lung homeostasis^9–11^. However, during lung carcinogenesis, there is aberrant expression of MUCs that promotes disease progression, serving as a hallmark for disease aggressiveness and worse outcomes^12–15^. MUCs are high-molecular-weight glycoproteins that are expressed by airway epithelial cells and are crucial for airway health and disease maintenance^10,11^. These MUCs are highly heterogeneous, O-glycosylated macromolecules characterized by the presence of several tandem repeats containing proline and numerous serine and/or threonine amino acid residues, the sites of O-glycosylation^10,12^. These mucins can be divided into two main families: secreted and cell-tethered mucins. The secreted mucins, i.e., MUC5AC and MUC5B, constitute a major component of the mucus layer, which is involved in airway homeostasis^12^. Any alterations in their expression, increases in their secretion, or alterations in their structure are associated with various pathological conditions and inflammation^9,12^. The aberrant overexpression of Muc5ac is also significantly associated with the development of lung adenocarcinoma (LUAD) mediated by a predisposition to Kras mutation^16^. MUC5AC, in conjunction with the membrane-tethered integrin β4, transmits molecular signals to induce cell migration by activating focal adhesion kinase in LC cells^13^. Furthermore, posttranslational modifications, such as the sialylation of MUC5AC by the enzyme ST6 *N*-acetylgalactosaminide alpha-2,6-sialyltransferase I (ST6GalNAc-I), significantly contribute to liver metastasis of LUAD^17^.

Despite the well-established role of MUC5AC in LC progression, metastasis, and survival, its specific contribution to the development of brain metastasis remains unclear. Therefore, this study aimed to elucidate the role of MUC5AC in the development of brain metastasis in LC patients.

## Materials and methods

### Cell lines, cell culture, and treatments

Brain-metastatic cell lines (A549-BrM, PC9-BrM3, and H1975-BrM) were generously gifted by Professors Patricia S. Steeg (National Cancer Institute) and Ann Marie Pendergast (Duke University School of Medicine) and cultured in RPMI 1640 medium supplemented with 10% fetal bovine serum (FBS) and penicillin/streptomycin antibiotic cocktail. Normal human astrocytes (HAs) were maintained in an astrocyte medium (Cat# 1801, ScienCell). The human microglial cell line HMC3 was maintained in EMEM supplemented with FBS. The cells were rinsed twice with phosphate-buffered saline (PBS) and exposed to an astrocyte-conditioned medium (ACM) at a 1:1 ratio for 24–48 h. For ligand treatments, cells were washed with PBS, cultured under low-serum conditions for 24 h, treated with the respective ligands for an additional 48 h in six-well plates, washed with PBS, and subjected to continued ligand treatment in 1–2% serum-containing growth medium for another 24 h. Proliferation assays were conducted using an IncuCyte system, and images were captured at 4-h intervals.

### Knockdown experiments

A cell line with stable MUC5AC knockdown (via pSUPER-Retro-shMUC5AC) was obtained after selection with puromycin (2 µg/ml) as previously described^13,18^. For transient knockdown of Annexin A2, ON-TARGETplus Human ANXA2 (302) siRNA-SMARTpool (#L-010741-00-0005, Dharmacon) was purchased. Transient transfection of siRNA was performed using TurboFect Transfection Reagent. The total lysate was collected in RIPA buffer for subsequent experiments.

### Culture supernatant collection

CM from astrocytes was obtained by replacing the medium with low-serum medium (1–2% FBS) for 48 h, collecting the CM, clearing it of debris by centrifugation at 5000 rpm for 5 min, filtering the supernatant through 0.45-μm filters, and storing it at −80 °C.

### Cytokine array

A cytokine array analysis was performed following the manufacturer’s instructions using culture supernatants collected under different culture conditions: medium alone, astrocytes alone, cancer cells alone, and cocultures of cancer cells and astrocytes (1:2 ratio). A Human Cytokine Array Panel A (Cat# ARY005B, R&D Systems, Inc.) was utilized for these experiments.

### Real-time PCR

Total RNA isolation and qRT-PCR were performed as previously described^17^. The primers used were as follows: h_MMP2-F 5’-AGCGAGTGGATGCCGCCTTTAA-3’, h_MMP2-R5’-CATTCCAGGCATCTGCGATGAG-3’, h_MMP9-F 5’-GCCACTACTGTGCCTTTGAGTC-3’, h_MMP9-R 5’-CCCTCAGAGAATCGCCAGTACT-3’, h_beta Actin-F 5’-CACCAACTGGGACGACAT, and h_beta Actin-R5’-ACAGCC TGGATAGCAACG-3’. Differences in gene expression are represented as fold changes (2^−ΔΔCT^).

### Western blotting

Whole-cell lysates were prepared as previously described^18–20^. Proteins in the lysates were separated on 8–12% SDS‒PAGE gels and transferred to PVDF membranes, and the membranes were blocked with 5% nonfat milk in PBS containing 0.1% Tween 20 (PBS-T) for 1 h at room temperature. After blocking, the membranes were washed with PBS-T and incubated with antibodies against the following proteins (1:1000 dilutions): MUC5AC (#MAB2011, Millipore Sigma), pAKT^S473^ (#927, Cell Signaling Technology), panAKT (#4691, Cell Signaling Technology), pERK1/2^T202/Y204^ (#4370, Cell Signaling Technology), ERK (#4695, Cell Signaling Technology), Integrin β4 (#14803, Cell Signaling Technology), pFAK^Y925^ (#3284, Cell Signaling Technology), pFAK^Y397^ (#3283, Cell Signaling Technology), FAK (#71433, Cell Signaling Technology), ZO1 (#13663, Cell Signaling Technology), N-cadherin (#13116, Cell Signaling Technology), Annexin A2 (#66035-1-Ig, Proteintech), SP1 (#sc-17824, Santa Cruz), fibronectin (#26836, Cell Signaling Technology), ZEB1 (#3396, Cell Signaling Technology), Slug (#9585, Cell Signaling Technology), tPA (#MA5-32507, Thermo Fisher), and β-actin (#4967, Cell Signaling Technology). Subsequently, the membranes were washed with PBS-T and incubated with either anti-mouse or anti-rabbit secondary antibodies conjugated to horseradish peroxidase (1:5000, Thermo Scientific) at room temperature for 1 h. The membranes were washed three times with PBS-T at room temperature, and signals were visualized using a chemiluminescence reagent.

### Transwell migration and invasion assays

A549-BrM.scr and A549-BrM5AC.sh1 cells (1 × 10^5^) were seeded on top of a Matrigel-coated insert in a Boyden chamber (8 μm pore size, Corning® BioCoat™ Matrigel® Invasion Chamber; # 354480) containing serum-free medium. For the migration assay, 0.5 × 10^5^ cells and a membrane without a Matrigel coating were used. The cells were seeded with or without a peptide inhibitor of ANXA2 (LCKLSL, 20 µM). RPMI 1640 medium containing ~20% FBS was added to each lower chamber of the 24-well plate. After 24 h, the cells remaining in the top chamber were carefully removed using a cotton swab, and the cells on the lower side of the membrane were fixed in methanol and stained with 0.2% (w/v) crystal violet. The number of migrated or invaded cells was counted in different fields of view (FOVs) at 10x magnification.

### Immunohistochemical analysis of tumor samples

We utilized commercially available tissue arrays (#GL861a and #LC812) from TissueArray and procured tissue blocks from PrecisionMed to analyze the expression of MUC5AC and Annexin A2.

Human tissue and mouse xenograft tissue slides were baked overnight in an oven at 58 °C, deparaffinized, and sequentially rehydrated through a graded alcohol series. Endogenous peroxidase activity was quenched with 3% hydrogen peroxide (methanol) for 1 h in the dark, and antigen retrieval was performed with 0.01 M citrate buffer (pH 6.0) containing 0.01% Tween 20 in a decloaking chamber (Biocare Medical). The slides were blocked with 10% normal horse serum solution (Vector Laboratories) for 1 h at room temperature and incubated with antibodies against MUC5AC (1:500, #MAB2011, Millipore Sigma), Annexin A2 (1:5000, #66035-1-Ig, Proteintech), Ki-67 (1:400, #9027, Cell Signaling Technology), and CK7 (1:300) overnight at 4 °C. The next day, the tissue sections were washed three times with PBS-T (5 min each) and incubated with a universal anti-rabbit/anti-mouse secondary antibody (Vector Laboratories) for 30 min at room temperature. Finally, the sections were washed with PBS-T (3X, 5 min), color was developed with a DAB substrate kit (Vector Laboratories), and the sections were counterstained with hematoxylin, dehydrated in a graded ethanol series, washed with xylene, and mounted with Permount (Vector Laboratories). Positive staining was assessed by an experienced pathologist and represented as the histoscore (H-score).

### Chromatin immunoprecipitation

Chromatin immunoprecipitation (ChIP) was conducted using a Pierce Agarose ChIP Kit (#26156) with minor modifications. Approximately 10 × 10^6^ cells with or without treatment were subjected to crosslinking with 1% formaldehyde for 10 min at room temperature. Crosslinking was terminated by incubation with 0.125 M glycine for 5 min, and the cells were then washed with chilled PBS (2X). The cells were then harvested by scraping, collected by centrifugation (3000 × *g*, 5 min, 4 °C), lysed, subjected to MNase digestion, and subjected to ChIP DNA enrichment with Sp1 (#9389, Cell Signaling Technology) and normal rabbit IgG. Quantitative real-time PCR using SYBR Green was performed on a CFX Connect Real-Time PCR Detection System (Bio-Rad Laboratories, Hercules, CA) for analysis. The primers utilized were SP1-CHIP F- 5’-TCTGGCTGAGGGAGGAGAAA-3’ and SP1-CHIP R- 5’-AGTCACTGGCCCAGTCCATA-3’. The ChIP gene fragments were quantified using a fold enrichment method based on the Ct value (2^−(Ct(Sp1) – Ct(IgG))).

### Bioinformatic analysis

Normalized gene expression analyses were performed on TCGA datasets extracted from the UCSC Xena Browser (https://xena.ucsc.edu/) and Firebrowse (https://firebrowse.org/) as of February 2022. Enrichment analyses were performed using a gene set enrichment tool (GSEA, https://www.gsea-msigdb.org/gsea/index.jsp).

### Proteomic analysis

Approximately 100 µg of protein per sample from three biological replicates per group was processed as follows. Chloroform/methanol extraction was used to remove detergent. The protein pellet was resuspended in 100 mM ammonium bicarbonate and digested with MS-grade trypsin (Pierce) overnight at 37 °C, which was followed by reduction with 10 mM DTT at 56 °C for 30 min and alkylation using 50 mM iodoacetamide at room temperature for 25 min. Peptides were cleaned with PepClean C18 spin columns (Thermo Scientific) and resuspended in 2% acetonitrile (ACN) and 0.1% formic acid (FA); 500 ng of each sample was loaded onto an Acclaim PepMap 100 75 µm × 2 cm C18 LC trap column (Thermo Scientific) at a flow rate of 4 µl/min and then separated on a Thermo Easy-Spray PepMap RSLC C18 75 µm × 50 cm C18 2 µm column (Thermo Scientific) in a Thermo RSLC Ultimate 3000 system (Thermo Scientific™) with a step gradient of 4–25% solvent B (0.1% FA in 80% ACN) from 10–100 min and 25–45% solvent B from 100–130 min at 300 nL/min and 50 °C with a 155 min total run time. The eluted peptides were analyzed by a Thermo Orbitrap Fusion Lumos Tribrid mass spectrometer (Thermo Scientific) in data-dependent acquisition mode. A full survey MS scan (from m/z 350–1800) was performed in the Orbitrap with a resolution of 120,000. The AGC target for MS1 was set to 4 × 10^5^, and the ion fill time was set to 100 ms. The most intense ions with charge states of 2–6 were isolated in 3 s cycles, subjected to HCD fragmentation with a normalized collision energy of 35%, and detected at a mass resolution of 30,000 at 200 m/z. For MS/MS, the AGC target was set to 5 × 10^4^, and the ion fill time was set to 60 ms; the dynamic exclusion was set to 30 s with a 10-ppm mass window. Each sample was run in duplicate. Protein identification was performed by searching the MS/MS data against the Swiss-Prot human protein database downloaded in July 2020 using the in-house PEAKS X + DB search engine. The search was set up for full tryptic peptides with a maximum of two missed cleavage sites. Acetylation of the protein N-terminus and oxidation of methionine were included as variable modifications, and carbamidomethylation of cysteine was set as a fixed modification. The precursor mass tolerance threshold was set to 10 ppm, and the maximum fragment mass error was set to 0.02 Da. The significance threshold of the ion score was calculated based on a false discovery rate of ≤1%. Quantitative data analysis was performed using Progenesis QI proteomics 4.2 (Nonlinear Dynamics).

### Interactome studies

For interactome studies, cancer cells were lysed in RIPA buffer supplemented with a protease inhibitor cocktail, and the protein concentrations in the lysates were quantified with a BCA protein assay. Approximately 400 μg of total cellular protein was subjected to immunoprecipitation (IP) with an anti-CLH2 antibody using Protein A/G agarose beads. IgG was used as a negative control. The IP samples eluted in 2X sample loading buffer were subjected to proteomic analysis by the UNMC proteomics core facility.

### Coimmunoprecipitation

Lysates from A549-BrM.scr and A549-BrM5AC.sh1 cells (500 μg) were incubated overnight with anti-MUC5AC (CLH2) and anti-ANXA2 antibodies (2 μg). Protein A/G agarose beads were added to the lysate-antibody mixture, and the mixture was incubated for 4 h at 4 °C in a rotary evaporator and then washed and eluted in 2X sample loading buffer. Proteins in the IP samples and input samples were electrophoretically separated on 2% SDS agarose gels. Proteins were transferred to membranes, which were blocked and then incubated with the appropriate antibodies. The signals were visualized using a chemiluminescence reagent.

### Animal studies

The Institutional Animal Care and Use Committees (IACUC) at the University of Nebraska Medical Center reviewed and approved all animal care and procedures. Approximately 1.0 × 10^6^ luciferase-labeled cells (A549-BrM.scr and A549-BrM5AC.sh) in 50–100 μl of PBS were injected into the left ventricle of 5–6-week-old athymic nude mice (*n* = 5 mice/group). The systemic distribution of cancer cells in different organs of the nude mice was assessed through bioluminescence imaging after intraperitoneal injection of D-luciferin (150 mg/kg). Beginning 7 days post-injection, the mice were examined for brain metastases weekly via bioluminescence imaging. Throughout the experimental period, the mice were monitored for signs of physical or neurological distress. At the experimental endpoint, the mice were euthanized, and tissues (brain, lung, liver, pancreas, spleen, and bone) were collected for further histopathological analysis.

### Statistical analysis

All in vitro experiments were independently performed at least three times. The statistical significance of differences between groups was determined using a two-tailed Student’s *t*-test. Statistical analyses were performed, and graphs were generated using GraphPad Prism (v9). A *p* value ≤0.05 was considered to indicate statistical significance.

## Results

### Depleting MUC5AC leads to decreased brain metastasis of non-small cell lung carcinoma

**Fig. 1:**
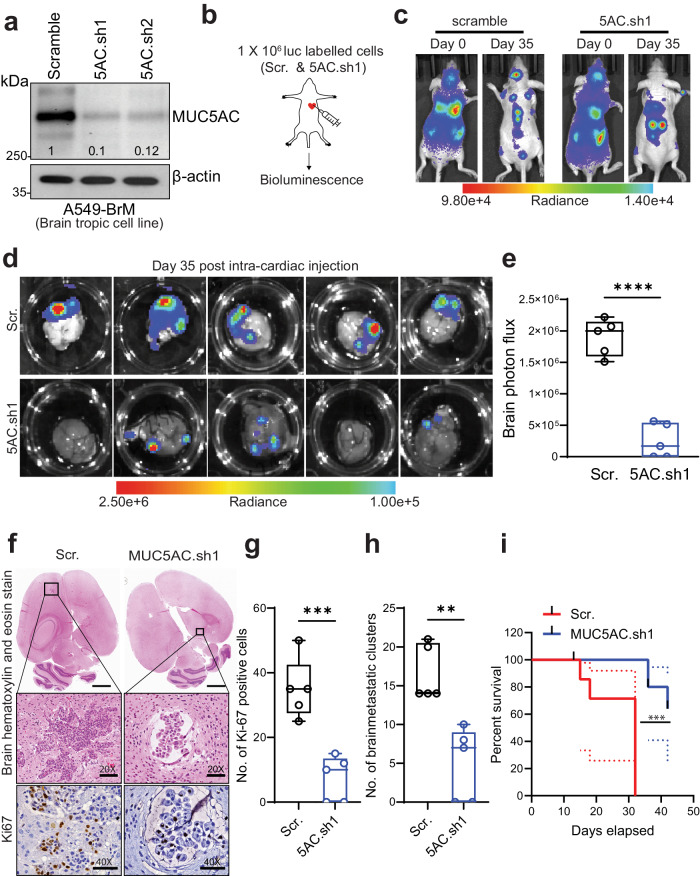
Knockdown of MUC5AC causes a significant reduction in brain metastasis in vivo. **a** Stable knockdown of MUC5AC in a brain-metastatic cell line (A549-BrM). Forty micrograms of total protein lysate were subjected to separation on a 2% agarose gel for 4 h at 100 V. β-Actin was used as an internal loading control. **b** Schematic of intracardiac injection in athymic nude mice (*n* = 5 mice/group, Scr. and MUC5AC KD). **c** Representative bioluminescence images of intracardially injected nude mice on Day 0 (D0) and Day 35 (D35). **d** Bioluminescence images of the mouse brain (Scr. and MUC5AC KD) on Day 35 after intracardiac injection. **e** Box plot of brain photon flux measured ex vivo after euthanasia of nude mice after D35. Mean ± SD (*n* = 5 mice/group). **f** Whole-brain hematoxylin and eosin images of scramble and MUC5AC KD cell-injected mice. Immunohistochemical analysis of Ki-67 staining in the brains of scramble and MUC5AC KD cell-injected mice. Mean ± SD (*n* = 5 mice/group). The bar represents 40X optical magnification. **g** Box plot of the number of Ki-67-positive cells in the brains of scramble and MUC5AC KD cell-injected mice. Mean ± SD (*n* = 5 mice/group). **h** Box plot of the total number of brain-metastatic clusters in the brains of MUC5AC KD and scramble cell-injected mice. Mean ± SD (*n* = 5 mice/group). **i** Kaplan‒Meier survival curves of MUC5AC-KD cell-injected mice and scramble control cell-injected mice. *P* < 0.05 was considered to indicate statistical significance. Nonparametric student’s *t*-test.

We and others have previously demonstrated the tumor-promoting effect of the mucin MUC5AC in lung adenocarcinoma and various other tumors^13,18,21^. However, the contribution of MUC5AC to brain metastasis of LUAD is currently unexplored. In this study, we performed stable knockdown (KD) of MUC5AC in brain-tropic cells (A549-BrM), and the efficacy of MUC5AC KD was confirmed at both the translational and transcriptional levels (Fig. 1a and Supplementary Fig. 1). To investigate the prometastatic role of MUC5AC in brain metastasis development, we injected ~1.0 × 10^6^ live luciferase-labeled scramble (A549-BrM.scr) and MUC5AC KD (A549-BrM5AC.sh1) cells into the left ventricle of athymic nude mice (*n* = 5 mice per group) (Fig. 1b). Bioluminescence imaging (BLI) conducted 5–10 min after tumor cell injection confirmed the systemic spread of tumor cells (A549-BrM.scr and A549-BrM5AC.sh1) in the mice on Day 0 (d0) (Fig. 1c). These mice were monitored weekly to assess the extent of brain metastasis. On d35, a significant reduction in brain metastasis was observed in mice injected with MUC5AC-KD cells (A549-BrM5AC.sh1) compared to those injected with scramble cells (A549-BrM.scr) (Fig. 1c). Ex vivo analysis of mouse brains at d35 further confirmed this reduction in BLI intensity in the A549-BrM5AC.sh1 group (*P* = 0.0003), suggesting decreased tumor cell metastasis to the brain (Fig. 1d, e). Immunohistochemical (IHC) analysis of the mouse xenograft tissues revealed a substantial decrease in staining for Ki-67 (a proliferation marker) in the MUC5AC KD group compared to the scramble group (Fig. 1f, g). Further analysis of brain xenografts revealed a significant decrease (*p* = 0.0058) in the total number of metastatic clusters in the A549-BrM5AC.sh1 group compared to the A549-BrM.scr group (Fig. 1h). In addition to decreased metastasis, the MUC5AC KD mice also exhibited prolonged survival compared to the mice injected with scramble cells (Fig. 1i). Additionally, we observed reduced metastasis to the lungs upon MUC5AC knockdown (Supplementary Fig. 2a, b).

### Expression of MUC5AC in primary NSCLC tumors and NSCLC brain metastases

**Fig. 2:**
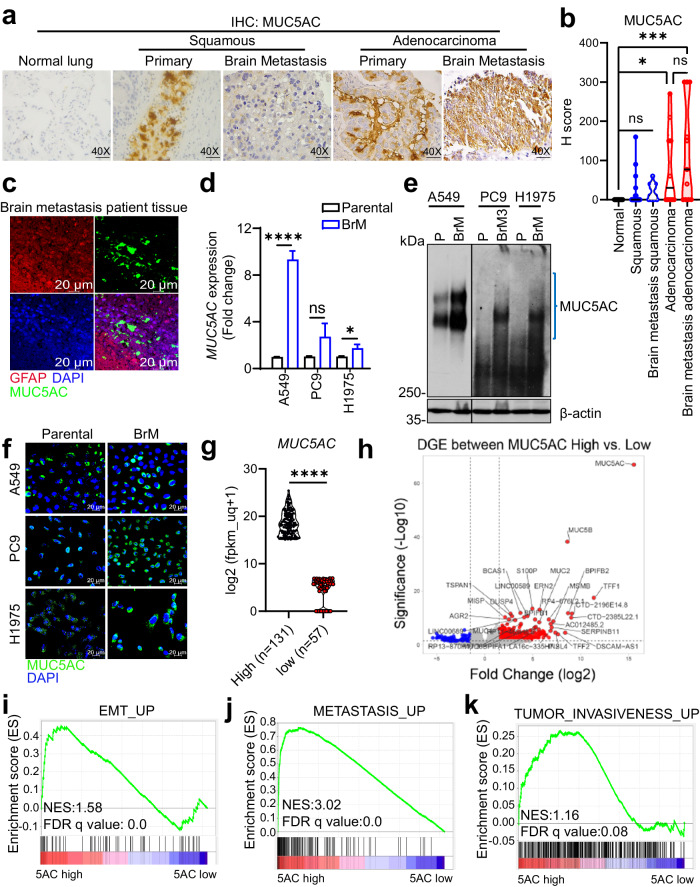
MUC5AC is highly expressed during brain metastasis in NSCLC. **a** Representative immunohistochemical images of MUC5AC staining with the clone 45M1 antibody in the normal lung (*n* = 10), lung adenocarcinoma (*n* = 10), lung squamous carcinoma (*n* = 29), and lung cancer brain metastasis (adenocarcinoma, *n* = 11; squamous cell carcinoma, *n* = 13) tissues. All the samples were unmatched. **b** Quantification of MUC5AC staining represented as the H-score. **c** Immunofluorescence staining of cancer cells and astrocytes for MUC5AC (green) and GFAP (red), respectively, in brain metastasis tissues. Nuclei were stained with DAPI. **d** Changes in the MUC5AC transcript level were quantified by qRT‒PCR and are presented as the fold changes (2^−ΔΔCt^). Actin was used as an internal control. Mean ± SD of *n* = 4 samples. *P* < 0.05 was considered to indicate statistical significance. Nonparametric Student’s *t-*test. **e** 2% agarose gel showing MUC5AC expression in brain-tropic (A549-BrM, PC9-BrM3, and H1975-BrM) and parental (A549, PC9, and H1975) cell lines. β-Actin was used as an internal control. **f** Immunofluorescence staining of MUC5AC in brain-metastatic and parental lung cancer cells. Nuclei were stained with DAPI. **g** Violin plot of MUC5AC expression in LUAD patients (TCGA-LUAD) divided into the high expression (*n* = 131) and low expression (*n* = 57) cohorts using the UCSC Xena Browser (https://xena.ucsc.edu/). **h** Volcano plots of differentially expressed genes between the MUC5AC-high and MUC5AC-low cohorts (https://xena.ucsc.edu/). **i**–**k** Overrepresentation analysis (GSEA) of differentially expressed genes (MUC5AC-high cohort versus MUC5AC-low cohort) in reference gene sets (GOTZMANN_EPITHELIAL_TO_MESENCHYMAL_TRANSITION_UP, ANG_TUMOR_INVASIVENESS_UP, WINNEPENNINCKX_MELANOMA_METASTASIS_UP).

As depletion of MUC5AC resulted in reduced brain metastasis, we sought to explore the status of MUC5AC expression in brain metastasis tissues compared to the primary lung tumors. IHC analysis with an anti-MUC5AC antibody (45M1 clone) was performed on normal (*n* = 10), primary lung adenocarcinoma (*n* = 10), primary lung squamous cell carcinoma (*n* = 29), squamous cell carcinoma brain metastasis (*n* = 13) and adenocarcinoma brain metastasis (*n* = 11) tissues. MUC5AC staining was notably stronger in the adenocarcinoma brain metastasis tissues than in the primary lung tumor tissues (Fig. 2a, b). Subsequent immunofluorescence (IF) staining of brain metastasis tissues using the brain-specific glial cell markers glial fibrillary acidic protein (GFAP) and MUC5AC (45M1) further confirmed elevated MUC5AC expression in brain metastasis tissues (Fig. 2c). We further analyzed MUC5AC expression in various brain-metastatic cell lines (A549-BrM, PC9-BrM3, and H1975-BrM) and their corresponding parental lines (A549, PC9, and H1975). Separation by 2% agarose gel electrophoresis, real-time reverse transcription PCR analysis, and immunofluorescence analysis revealed a significant increase in the MUC5AC level in the brain-tropic cell lines compared to their parental cells (Fig. 2d–f).

Given the significant increase in MUC5AC expression in LUAD patients, we analyzed the differentially expressed genes (DEGs) between patients with high MUC5AC expression and those with low MUC5AC expression in the TCGA-LUAD cohort (Fig. 2g, h). Subsequent gene set enrichment analysis (GSEA) of the DEGs in the MUC5AC-high compared with the MUC5AC-low patient groups revealed enrichment of gene signatures associated with epithelial-to-mesenchymal transition (EMT), increased tumor invasiveness, and increased metastasis, suggesting the potential involvement of MUC5AC in LUAD metastasis (Fig. 2i–k).

### Global proteomic analysis reveals the impact of MUC5AC on the metastatic phenotype of brain-metastatic cells

**Fig. 3:**
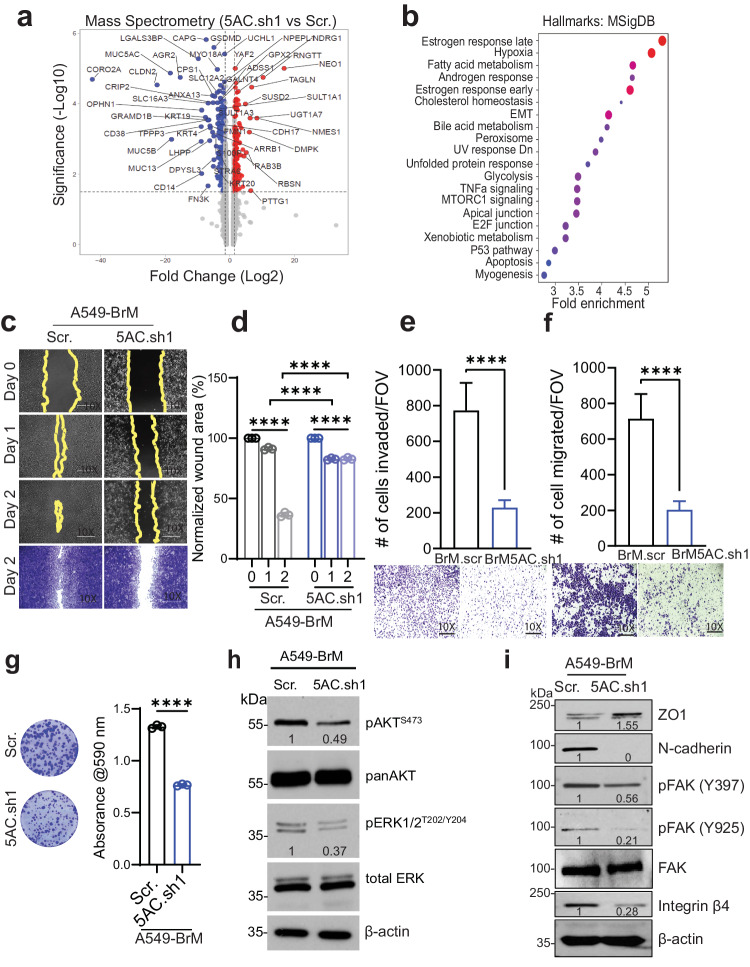
MUC5AC maintains mesenchymal cell polarity by affecting several cell adhesion molecules. **a** Volcano plot of differentially expressed proteins in the MUC5AC-depleted (5AC.sh1) and scramble (Scr.) brain-tropic cells (A549-BrM). **b** Enrichment analysis (Hallmarks: MSigDB) of differentially expressed proteins in MUC5AC-KD and scramble cells. **c**, **d** Wound healing assay results showing a significant reduction in the wound closure ability of MUC5AC-knockdown (A549-BrM5AC.sh1) cells compared to that of A549-BrM.scr cells at different time points: Days 0–2 (D0–D2). On D2, the cells were stained with 5% crystal violet and imaged. The invasion (**e**) and migration (**f**) of MUC5AC-depleted brain-tropic cells was reduced compared to that of the scramble control cells. **g** Significant reduction in the clonogenic growth of MUC5AC-knockdown cells (A549-BrM5AC.sh1) compared to their A549-BrM.scr counterparts. **h** Western blot analysis of the proliferation markers phosphorylated AKT (S473) and ERK (T202/Y204) and the corresponding total AKT and ERK protein levels. **i** Western blot analysis of several metastasis-associated proteins in MUC5AC KD and scramble A549-BrM cells. β-Actin was used as an internal control. *P* < 0.05 was considered to indicate statistical significance. Mean ± SD. Nonparametric Student’s *t-*test.

MUC5AC is known to activate EMT and transwell migration in various types of tumor cells^13,22^. In our study, the transwell migration and wound healing potential of brain-metastatic cells with high MUC5AC expression were significantly increased compared with those of the parental cells (Supplementary Fig. 3a, b). Further analysis also revealed the upregulation of several EMT markers in the metastatic lines compared to the control parental lines (Supplementary Fig. 3c). To comprehensively understand the global impact of MUC5AC on the metastatic phenotype of non-small cell lung cancer (NSCLC) cells, we conducted mass spectrometry (MS) analysis on total lysates of MUC5AC-KD (A549-BrM-5AC.sh1) and scramble (A549-BrM.scr) cells. The analysis revealed the downregulation of 304 proteins and the upregulation of 91 proteins in MUC5AC KD cells. Some of the key downregulated proteins included MUC5AC, CORO2A, CLDN2, LGALS38P, and AGR2 (Fig. 3a). Enrichment analysis of these differentially expressed proteins (1.5-fold up- or downregulated) in MUC5AC KD cells demonstrated significant enrichment of proteins associated with the hallmarks of the EMT phenotype (Fig. 3b). Furthermore, wound healing, invasion and transwell migration assays demonstrated a substantial reduction in the migratory capacity of MUC5AC KD cells compared to that of scramble control cells (Fig. 3c–f). Additionally, compared with control cells, MUC5AC KD (A549-BrM5AC.sh1) cells exhibited decreased clonogenicity (Fig. 3g) and reduced expression of proliferation markers, such as phosphorylated AKT (S473) and ERK (T202/Y204) (Fig. 3h). Western blot analysis of the total lysates further confirmed the downregulation of certain markers of metastasis, such as N-cadherin, integrin β4, and phosphorylated FAK (Y397, Y925), and the upregulation of the epithelial marker ZO1 in MUC5AC-KD cells compared to scramble control cells (Fig. 3i). These findings collectively indicate that the induction of metastatic behaviors of LC cells is mediated by MUC5AC expression.

### Global interactome analysis reveals the cooperation of MUC5AC with annexin A2 during brain metastasis

**Fig. 4:**
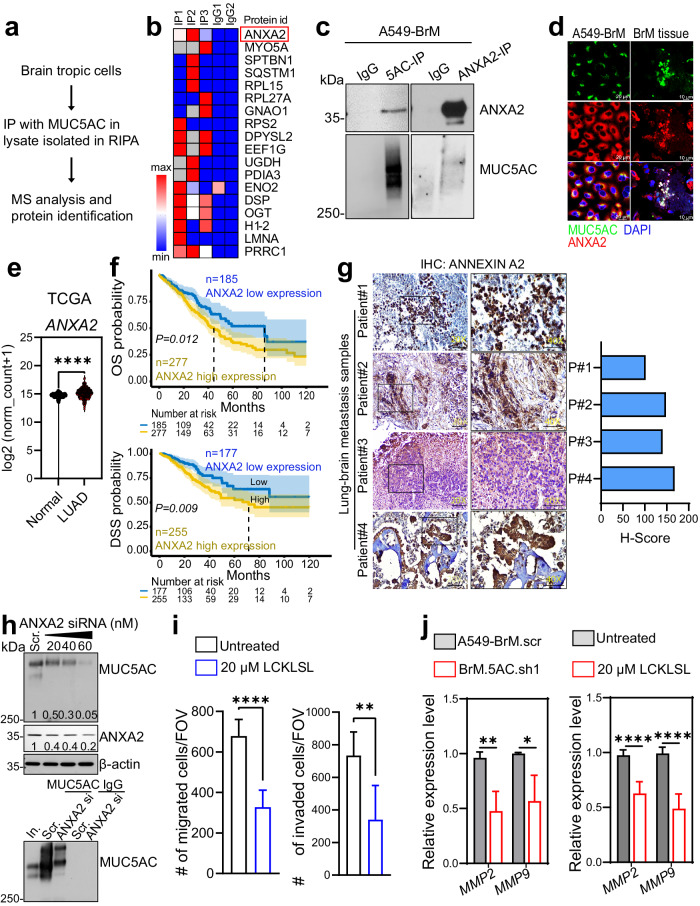
MUC5AC interacts with Annexin A2. **a**, **b** Workflow and LC‒MS/MS analysis of the MUC5AC-interacting partners identified after immunoprecipitation with an anti-MUC5AC antibody (clone CLH2). **c** Western blot analysis results confirming the physical interaction between MUC5AC and ANXA2 via IP and reciprocal IP assays. **d** Immunofluorescence analysis of MUC5AC (488 nm) and ANXA2 (568 nm) in A549 cells and BrM tissues. **e** Violin plot of ANXA2 expression in primary tumor samples represented in TCGA-LUAD (*n* = 515) compared to that in normal lung samples in GTEx (*n* = 288). **f** Kaplan‒Meier survival curve (TCGA-LUAD) showing a significant reduction in overall survival (OS) and disease-specific survival (DSS) in patients with high ANXA2 expression compared to patients with low ANXA2 expression (https://tau.cmmt.ubc.ca/cSurvival/). **g** Immunohistochemical analysis of ANXA2 in lung adenocarcinoma brain metastasis tissues from patients. **h** Analysis of MUC5AC in total lysates from A549-BrM cells with transient ANXA2 knockdown by 2% agarose gel electrophoreses. β-Actin was used as an internal control (upper panel). Analysis of MUC5AC-immunoprecipitated samples (clone CLH2) from the scramble and ANXA2 siRNA (40 nM) cell lysates by 2% agarose gel electrophoresis. **i** Transwell migration and invasion assays of A549-BrM cells after treatment with the ANXA2 peptide inhibitor LCKLSL (20 µM) for 24 h. **j** Real-time RT‒PCR analysis of MMP2 and MMP9 expression in MUC5AC KD cells compared to scramble control cells. The expression of these genes was further analyzed in cells treated with 20 µM LCKLSL or DMSO as the control. *P* < 0.05 was considered to indicate statistical significance. Mean ± SD. Nonparametric Student’s *t*-test.

Due to the secretory nature of MUC5AC, it may interact with a membrane-bound protein to exert its functional effect during the metastasis process. To identify its interacting molecules, we conducted an unbiased approach using LC‒MS/MS analysis of proteins that coimmunoprecipitated with MUC5AC from A549-BrM cells (Fig. 4a and Supplementary Fig. 4). IP was performed on whole-cell lysates using an anti-MUC5AC antibody (clone CLH2) and IgG, revealing ~18 novel proteins that coimmunoprecipitated with MUC5AC and suggesting potential partners associated with signaling and metastasis (Fig. 4b). We also validated these findings through IP and reciprocal IP in brain-metastatic cell lysates with anti-MUC5AC and anti-Annexin A2 (ANXA2) antibodies, confirming a physical interaction between these two proteins (Fig. 4c). Immunofluorescence analysis further supported these results, showing the colocalization of MUC5AC (green) and ANXA2 (red) in A549 cells and brain metastasis tissue (Fig. 4d).

Given the colocalization of and physical interaction between MUC5AC and ANXA2, we investigated the function of ANXA2 in LUAD progression. Analysis of the TCGA dataset revealed significant upregulation of ANXA2 in LUAD tissue samples compared to normal tissue samples (Fig. 4e). Additionally, at the clinical level, high ANXA2 expression was also correlated with worse overall survival (*P* = 0.012) and disease-specific survival (*P* = 0.009) in LUAD patients compared with those of patients with low ANXA2 expression (Fig. 4f). IHC analysis also revealed elevated ANXA2 expression in LUAD brain metastasis tissues (Fig. 4g). Furthermore, colocalization experiments with anti-MUC5AC (CLH2) and anti-tissue plasminogen activator (tPA) antibodies demonstrated strong colocalization, suggesting the potential involvement of the MUC5AC/ANXA2/tPA signaling axis during LUAD brain metastasis (Supplementary Fig. 5).

To explore the significance of the MUC5AC–ANXA2 interaction during brain metastasis, we sought to determine the status of MUC5AC expression in ANXA2-depleted brain-tropic cells. Interestingly, 2% agarose gel electrophoresis of the total lysates and IP samples showed a reduction in the MUC5AC level upon transient silencing of ANXA2 in A549-BrM cells (Fig. 4h), possibly because of an effect on protein stability. Furthermore, when A549-BrM cells were pretreated with a competitive peptide inhibitor of ANXA2 (LCKLSL, 20 µM) for 36 h, there were significant reductions in cell migration and invasion, as shown by the results of the transwell migration and invasion assays in Boyden chambers (Fig. 4i). Additionally, real-time RT‒PCR analyses indicated decreased expression of MMP2 and MMP9 in both the LCKLSL-treated and MUC5AC KD cells compared to the controls, suggesting that MUC5AC/ANXA2 mediate metastasis by activating matrix metalloproteinases (Fig. 4j).

### The brain microenvironment supports the colonization of MUC5AC-expressing lung cancer cells during brain metastasis

**Fig. 5:**
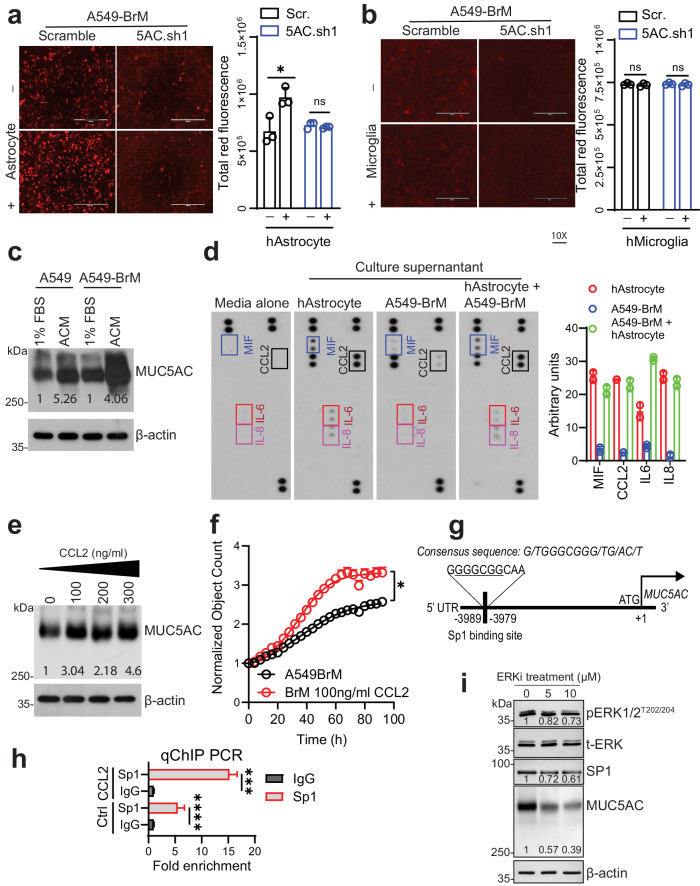
Astrocyte-derived CCL2 induces MUC5AC expression in the brain microenvironment. **a**, **b** Total cellular fluorescence of cancer cells (scramble and MUC5AC KD cells) after coculture with astrocytes and microglia (1:2) respectively, for 48 h. **c** 2% agarose gel electrophoresis was performed to detect MUC5AC after treatment of A549 and A549-BrM cells with astrocyte-conditioned medium (ACM) **d** Cytokine array analysis of culture supernatants collected from cultures of human astrocytes (HAs) alone, A549-BrM cells alone, HAs + A549-BrM cells (2:1 ratio), and medium alone. HAs strongly secreted MIF, CCL2, IL-6, and IL-8. **e** 2% agarose gel electrophoresis showed a concentration (100–300 ng/ml)-dependent increase in MUC5AC expression compared to that in the control group. **f** The growth kinetics of A549-BrM cells after 100 ng/ml CCL2 treatment were compared to those of control cells. **g** Schematic showing the Sp1 binding site in the 5’-UTR of the MUC5AC gene. **h** ChIP-quantitative PCR analysis of Sp1 binding sites in the MUC5AC promoter. The data were presented as the fold enrichment values. **i** Western blot results showing the effect on MUC5AC and SP1 expression after treatment with an ERK inhibitor (PD98059) for 36 h. β-Actin was used as a loading control. *P* < 0.05 was considered to indicate statistical significance. Mean ± SD. Nonparametric *t*-test.

The brain microenvironment contains astrocytes and microglia, which play crucial roles in supporting the colonization of tumor cells in the brain^2,23^. To understand the influence of the brain microenvironment on LC cell growth, we cocultured scramble and MUC5AC KD cells (A549-BrM.scr and A549-BrM5AC.sh1) with normal human astrocytes (NHAs) or microglia (HMC3) at a 1:2 ratio for 24–48 h. The coculture experiments revealed a significant increase in the total cellular fluorescence of A549-BrM.scr cells compared to that of the corresponding MUC5AC KD cells when cocultured with NHAs (Fig. 5a). However, there was no observable difference in fluorescence between the A549-BrM.scr and A549-BrM5AC.sh1 cells in the presence and absence of HMC3 cells, indicating the proliferative effect of NHAs on MUC5AC-expressing LC cells (Fig. 5b).

Subsequently, we treated the parental and BrM cells with conditioned medium collected from NHAs (ACM) cultured in 1% serum for 24–48 h. Analysis of the total lysates by 2% agarose gel electrophoresis revealed a 4–5-fold increase in MUC5AC expression in the ACM treatment group compared to the complete medium (1% serum) treatment group (Fig. 5c). As astrocytes and ACM influence the growth of LC cells and MUC5AC expression, respectively, we investigated soluble components released by astrocytes. A cytokine array analysis of conditioned medium (CM) collected from NHAs, A549-BrM cells, or A549-BrM+NHA (1:2) cells and medium alone revealed the release of key soluble factors, including MIF, CCL2, IL-6, and IL-8, from NHAs (Fig. 5d). Therefore, we treated cancer cells with the CCL2 ligand (100–300 ng/ml) for 24–48 h and found that MUC5AC expression was increased in A549-BrM cells in a dose-dependent manner (Fig. 5e). We further investigated the effect of other cytokines/chemokines, such as IL-6 and IL-8, at various concentrations (50–200 ng/ml); however, we found no observable difference in MUC5AC expression after 48 h of treatment (Supplementary Fig. 6). Additionally, a significant increase in cancer cell proliferation compared to that in the untreated control group was observed after treatment with 100 ng/ml CCL2 (Fig. 5f). To further investigate the mechanism by which CCL2 influences MUC5AC expression, we conducted an *in-silico* analysis of the 5’-untranslated region (UTR) and revealed the presence of a putative Sp1 transcription factor-binding site at nucleotides −3989 to −3979 in the MUC5AC gene (Fig. 5g). To further confirm this binding site, we performed chromatin immunoprecipitation with a ChIP-grade anti-Sp1 antibody and revealed a fourfold increase in the binding of Sp1 to the MUC5AC promoter in cells treated with CCL2 than in untreated controls (Fig. 5h). As CCR2, the cognate receptor for CCL2, acts via ERK signaling, A549-BrM cells were treated with the ERK inhibitor PD98059 (5 and 10 µM) for 48 h. Western blot analysis indicated dose-dependent decreases in the expression levels of MUC5AC and the transcription factor SP1 after PD98059 treatment compared to those in the DMSO control group (Fig. 5i). These findings suggest that CCL2 secreted by astrocytes induces MUC5AC expression via the ERK1/2-SP1 signaling pathway.

## Discussion

The lack of effective therapeutic options for LUAD patients with brain metastasis remains a significant concern^2,24–26^. Accumulating evidence suggests that the clonal expansion of specific cells in these tumors provides a favorable environment for their survival and colonization within the brain microenvironment^27–29^. Previous studies, including ours, have implicated aberrant MUC5AC overexpression in the progression and poor prognosis of LUAD^13,15–17^. Notably, a study by Bauer et al. ^16^ demonstrated the requirement of Muc5ac in *Kras*-driven lung tumors, further highlighting its potential as a determining factor in lung tumor prognosis^16^. Although the role of MUC5AC in maintaining lung homeostasis is well established^10,11^, its involvement in brain metastasis remains unknown. Our current study revealed that MUC5AC interacts with ANXA2 on the plasma membrane to facilitate metastasis and colonization in the brain microenvironment through CCL2 from astrocytes.

MUC5AC is normally expressed by airway epithelial cells in response to stresses such as exposure to cigarette smoke and allergens; however, it is also highly overexpressed in several cancers, including LUAD, potentially contributing to *Kras* mutant-driven LC^9–11,16,30^. Previous research has shown that MUC5AC interacts with transmembrane adhesion molecules, such as integrin β4, in LUAD cell lines and genetically engineered mouse models, resulting in the induction of migration in vitro^13^. MUC5AC also targets focal adhesion kinase (FAK) and its downstream molecules, including integrins (integrin β3, β4, and β5), promoting increased proliferation and invasion of metastatic LC cells^13^. Therefore, understanding whether these signaling pathways are associated with brain metastases is crucial. Previous studies have shown increases in the protein levels of metastasis-associated markers, such as integrin β4, pFAK (Y397), pFAK (Y925), and MUC5AC, in brain-metastatic cell lines^13,31^. MUC5AC exerts its effects by interacting with various transmembrane-associated proteins, including CD44, integrin β4, and integrin β5, during different tumor events^13,18,21^. However, the dependence of MUC5AC on these protein partners during brain metastasis remains unclear. Therefore, we conducted an interactome analysis after IP with MUC5AC in total lysates from a brain-metastatic cell line, revealing the presence of a strong physical interaction of MUC5AC with ANXA2, among other binding partners.

ANXA2 is a calcium-dependent phospholipid plasma membrane-binding protein that is upregulated in several tumors, including lung tumors^32,33^. It is also known to contribute to increased angiogenesis and extracellular matrix degradation. It also serves as a coreceptor for tissue plasminogen activator (tPA) and plasminogen, leading to tPA-dependent plasmin generation and metastasis^32^. Secretory ANXA2 in breast tumor serum creates a favorable environment for breast cancer cell survival and colonization in the brain through the activation of MAPK, NF-κB, and STAT3 signaling in macrophages, which results in the production of the chemokines IL-6 and TNFα^34^. We demonstrated the cooperation between ANXA2 and tPA along with MUC5AC in extracellular matrix (ECM) remodeling during the metastasis process. ECM remodeling is a pivotal step for cancer metastasis^35^. MUC5AC, with the help of ANXA2, promotes ECM degradation by activating matrix metalloproteinases (MMPs) during the brain metastasis of LUAD. Additionally, this degradation can be reversed by depleting MUC5AC or inhibiting ANXA2 activity with a peptide inhibitor.

**Fig. 6:**
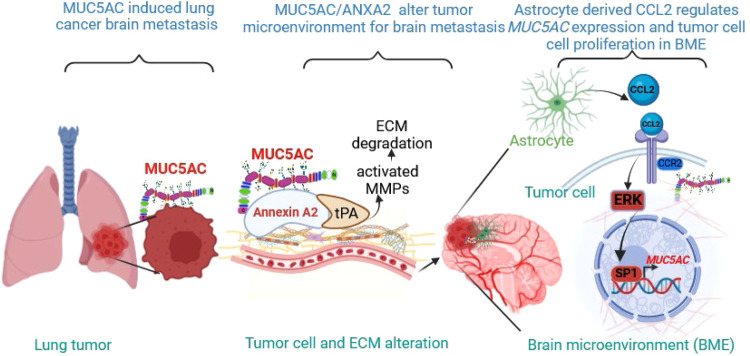
Model depicting the proposed mechanism of brain metastasis mediated by MUC5AC. During brain metastasis, MUC5AC interacts with ANXA2. As a result of the MUC5AC/ANXA2/tPA interaction, MMPs (MMP2 and MMP9) are activated, causing lung cancer cells to metastasize to the brain. The brain microenvironment, particularly the soluble factor CCL2 released from astrocytes, further increases the expression of MUC5AC, which facilitates tumor cell colonization in the brain.

The brain microenvironment, although immunosuppressive, contains other specific resident brain cells that can hinder tumor cell colonization and growth^29^. In certain tumors, such as NSCLC and breast cancer, glial cells can assist tumor cells in colonizing brain tissue and forming brain metastases^23^. Our evaluation with astrocytes and microglia revealed significant upregulation of MUC5AC expression upon treatment with ACM but no changes upon treatment with conditioned medium from microglia, suggesting the existence of paracrine communication between tumor cells and astrocytes. Glial cells also secrete factors that facilitate invasion, metastasis, and tumor cell colonization^23,36^. Thus, we analyzed the secretory factors in cultures of astrocytes and tumor cells and cocultures of these cells and found increased abundances of various soluble factors, such as MIF, CCL2, IL-6, and IL-8, primarily in astrocyte cultures compared to cultures of the other cell types. We also observed that treatment with ACM or CCL2, via the CCR2 axis, promoted cell proliferation by upregulating MUC5AC expression. However, there were no significant changes in cancer cell proliferation when MUC5AC was knocked down, and the cells were cocultured with astrocytes. Previous studies have demonstrated the importance of the CCL2–CCR2 pathway in regulating the brain metastasis of breast cancer by increasing blood‒brain barrier permeability^37^. This pathway also regulates cancer cell survival and motility by activating mitogen-activated protein kinase (MAPK) signaling and thus increasing ERK1/2 phosphorylation^38^. We demonstrated that disruption of MAPK signaling with an ERK inhibitor (PD98059) reduced MUC5AC expression via the transcription factor SP1 (Fig. 6). These data suggest that astrocyte-secreted CCL2, which modulates MUC5AC expression, is a major contributor to brain colonization in LUAD patients.

In conclusion, our study demonstrates, for the first time, the involvement of MUC5AC in brain metastasis via interaction with ANXA2 and the promotion of cell colonization in the brain microenvironment facilitated by the astrocyte-secreted chemokine CCL2 in LUAD.

### Supplementary information

Supplementary Information
