# Supplementary material for: Dissecting the MUC5AC/ANXA2 signaling axis: implications for brain metastasis in lung adenocarcinoma
Source: Exp Mol Med. 2024 Jun 3;56(6):1450–60. doi: 10.1038/s12276-024-01255-6 (PMC11263355; doi:10.1038/s12276-024-01255-6)
Supplement: Supplementary file 1 — Supplementary Information [file 12276_2024_1255_MOESM1_ESM.pdf]

Supplementary Fig. 1

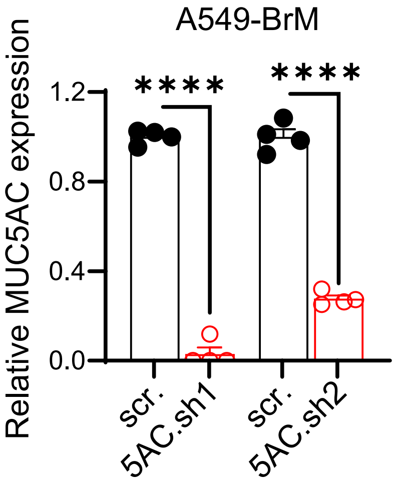

Supplementary Fig. 1. Generation of stable MUC5AC knockdown cells. qRT-PCR shows a knockdown of MUC5AC in the A54-BrM5AC.sh cells compared to scramble control. The data were represented as fold change (ΔΔCt). $P<0.05$ was considered statistically significant. Nonparametric Student's *t*-test.

Supplementary Fig. 2

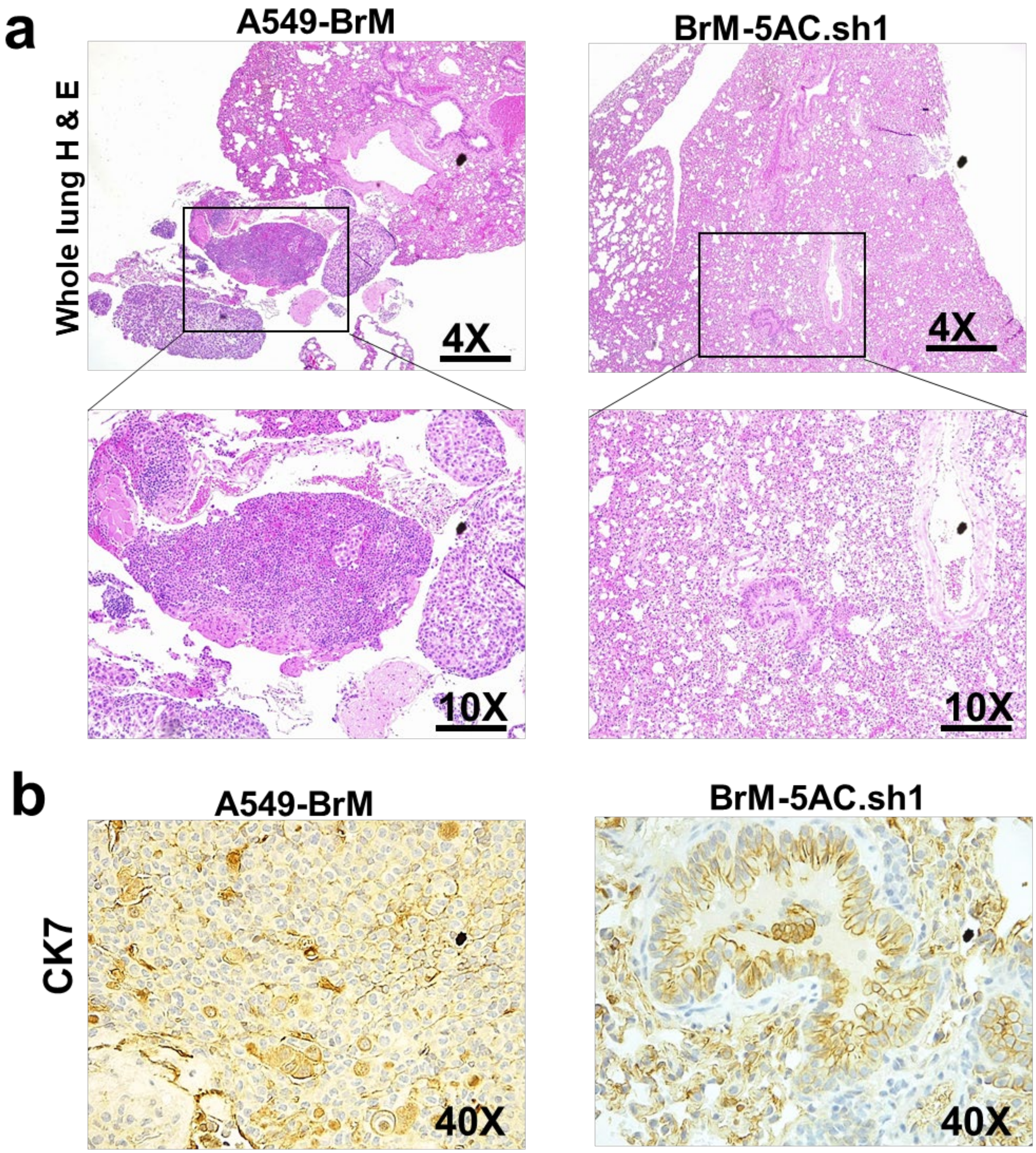

Supplementary Fig. 2. Intracardiac injection of MUC5AC-depleted cells shows decreased lung metastasis. a. Hematoxylin and eosin (H&E) staining of lung tissue from athymic nude mice after intracardiac injection. **b.** Decreased CK7 expression in the lungs of A549-BrM5AC.sh injected group cells compared to the lungs of the A549-BrM.scr group.

Supplementary Fig. 3

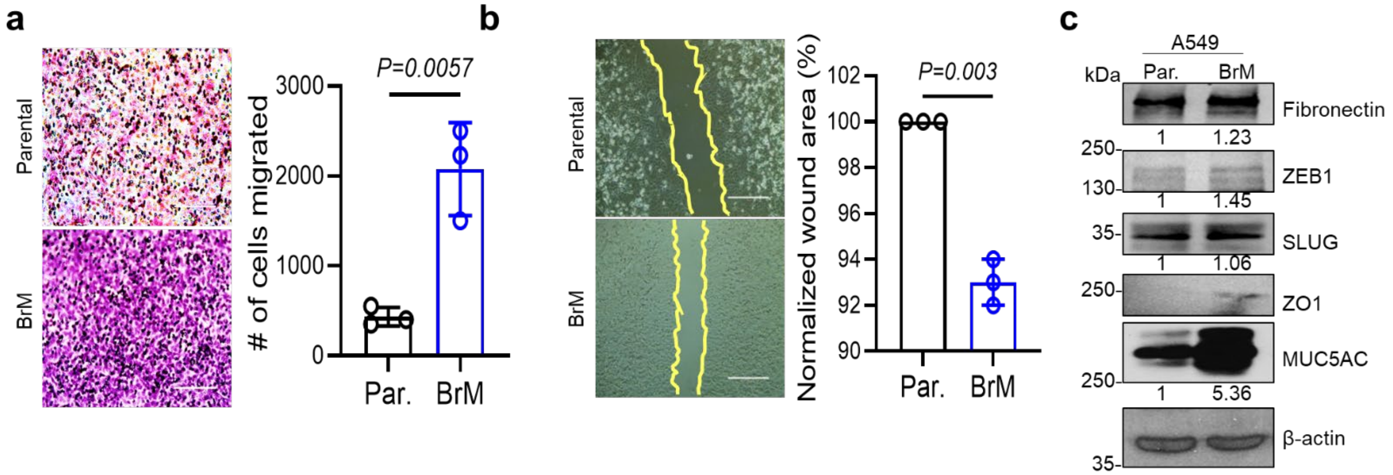

Supplementary Fig. 3. EMT phenotype and markers in brain tropic cell lines. a & b. Increased cell migration and wound healing of brain tropic cells (A549-BrM) compared to the parental line (A549). **c.** Western blots of epithelial to mesenchymal markers in the brain metastatic and parental lung cancer cell line. β-actin was used as an internal control. *P<0.05* was considered statistically significant.

Supplementary Fig. 4

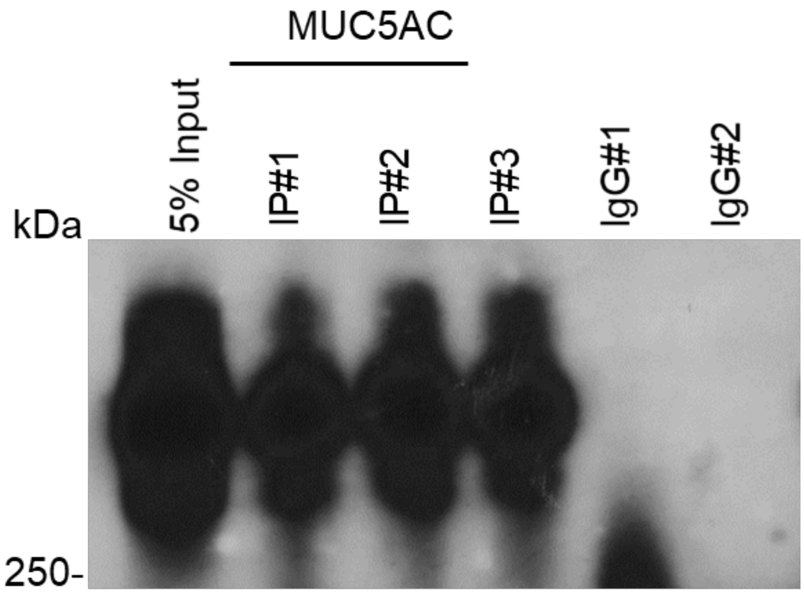

Supplementary Fig. 4. Immunoprecipitation of MUC5AC in the brain metastatic cell line. Immunoprecipitation with MUC5AC antibody (CLH2 clone) was performed in the A549-BrM cells in triplicates and respective IgG controls (duplicates). The immunoprecipitation of MUC5AC was confirmed in the 2% agarose gel.

Supplementary Fig. 5

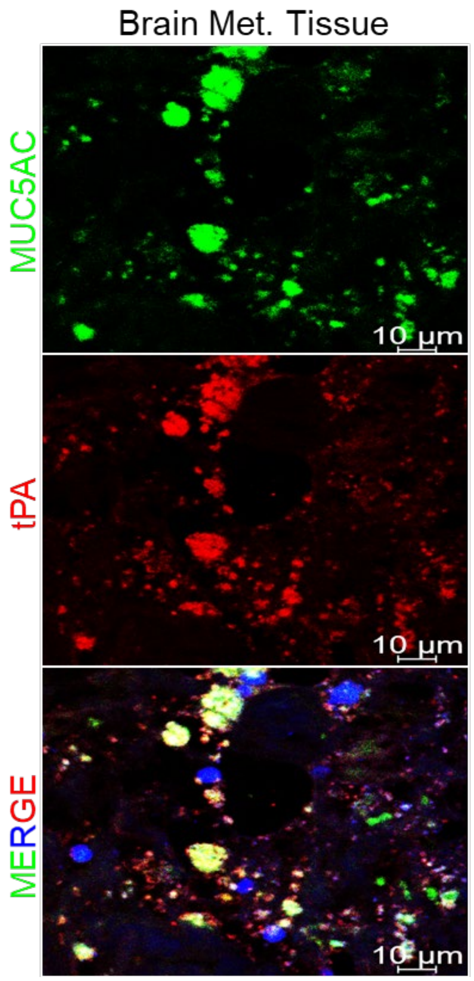

Supplementary Fig. 5. Colocalization of MUC5AC and tissue plasminogen activator (t-PA) in brain metastasis cells. a. Confocal microscopy of t-PA (red) and MUC5AC (green) in the LUAD-brain metastasis tissues.

Supplementary Fig. 6

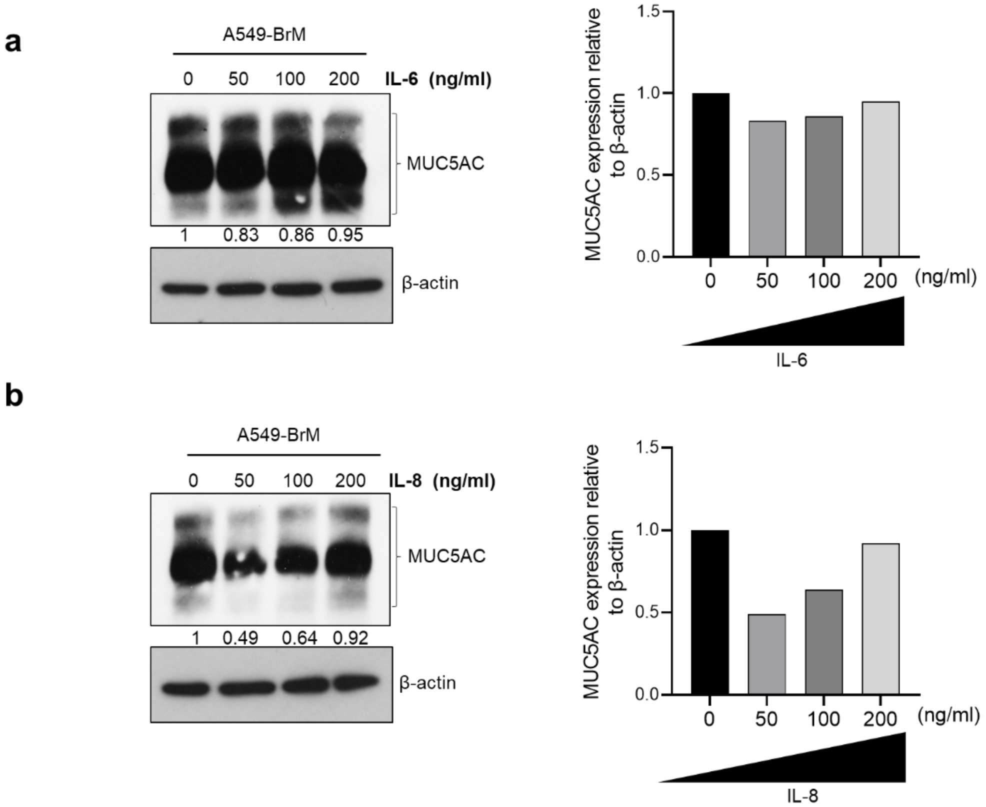

Supplementary Fig. 6. Effect of chemokines in the MUC5AC expression. a & **b**. MUC5AC expression after treatment with various doses of chemokines (IL-6 and IL-8) for 48h. β-actin was used as an internal control.
